# Development of Engineered-U1 snRNA Therapies: Current Status

**DOI:** 10.3390/ijms241914617

**Published:** 2023-09-27

**Authors:** Mariana Gonçalves, Juliana Inês Santos, Maria Francisca Coutinho, Liliana Matos, Sandra Alves

**Affiliations:** 1 Research and Development Unit, Department of Human Genetics, National Institute of Health Doutor Ricardo Jorge, INSA I.P., Rua Alexandre Herculano, 321, 4000-055 Porto, Portugal; mariana.goncalves@insa.min-saude.pt (M.G.); juliana.santos@insa.min-saude.pt (J.I.S.); francisca.coutinho@insa.min-saude.pt (M.F.C.); liliana.matos@insa.min-saude.pt (L.M.); 2 Center for the Study of Animal Science, Institute of Sciences, Technologies and Agro-Environment, CECA-ICETA, University of Porto, Praça Gomes Teixeira, Apartado 55142, 4051-401 Porto, Portugal; 3 Associate Laboratory for Animal and Veterinary Sciences, AL4AnimalS, Faculty of Veterinary Medicine, University of Lisboa, Avenida da Universidade Técnica, 1300-477 Lisboa, Portugal; 4 Centre for the Research and Technology of Agro-Environmental and Biological Sciences, CITAB, Inov4Agro, University of Trás-os-Montes and Alto Douro, 5000-801 Vila Real, Portugal; 5 Biology Department, Faculty of Sciences, University of Porto, Rua do Campo Alegre, 4169-007 Porto, Portugal

**Keywords:** U1 snRNA (small nuclear RNA)-based therapies, modified U1 snRNAs, exon-specific U1 snRNAs (ExSpeU1s), 5′ splice-site (5′ss), splicing mutations, splicing correction

## Abstract

Splicing of pre-mRNA is a crucial regulatory stage in the pathway of gene expression. The majority of human genes that encode proteins undergo alternative pre-mRNA splicing and mutations that affect splicing are more prevalent than previously thought. Targeting aberrant RNA(s) may thus provide an opportunity to correct faulty splicing and potentially treat numerous genetic disorders. To that purpose, the use of engineered U1 snRNA (either modified U1 snRNAs or exon-specific U1s—ExSpeU1s) has been applied as a potentially therapeutic strategy to correct splicing mutations, particularly those affecting the 5′ splice-site (5′ss). Here we review and summarize a vast panoply of studies that used either modified U1 snRNAs or ExSpeU1s to mediate gene therapeutic correction of splicing defects underlying a considerable number of genetic diseases. We also focus on the pre-clinical validation of these therapeutic approaches both in vitro and in vivo, and summarize the main obstacles that need to be overcome to allow for their successful translation to clinic practice in the future.

## 1. Introduction

In most eukaryotic organisms, genes are transcribed as pre-messenger RNAs (pre-mRNAs) that contain both non-coding sequences, introns, and coding sequences, exons. During the splicing process, introns are removed, leaving just exons in the so-called mature messenger RNA (mRNA). The mature mRNA is then transferred to the cytoplasm and translated into proteins [1]. Briefly, the splicing of pre-mRNA(s) is carried out by a complex known as spliceosome, which is constituted by five small nuclear ribonucleoproteins (snRNPs): U1, U2, U4, U5, and U6, together with more than 150 other proteins. The spliceosome recognizes the intron-exon junctions and its function is to remove the introns in order to form a mature in-frame mRNA which may then be translated into a protein [2,3,4].

Overall, splicing is a very complex step in the mRNA processing which involves a variety of factors. The recognition of exon/intron boundaries for correct intron removal by the splicing machinery requires the presence of several sequence elements on pre-mRNA, including the 5′ splice-site (ss) and 3′ss, the branch point sequence (BPS), and the polypyrimidine (Py) tract. In addition to these core splice-site motifs, other *cis*-regulatory elements that recruit specific RNA-binding proteins that either activate or repress the use of adjacent splice-sites contribute to the fine-tuning and specificity of this pre-mRNA processing event [5,6].

It is estimated that about 15% of all point mutations that result in genetic diseases in humans are caused by changes either in classical or auxiliary splicing sequence elements. Approximately 10% of those mutations affect 5′ and 3′ss, resulting most frequently in partial or whole exon skipping, and to a lesser extent in intron retention and cryptic splice-site activation [7,8,9]. Particularly, with respect to mutations affecting the 5′ss, these can reduce complementarity with the U1 small nuclear RNA (U1 snRNA), thus preventing spliceosome formation [10,11,12,13]. In fact, it is well-established that it is the U1 snRNP that initiates spliceosome assembly by binding to the 5′ss. This binding relies on a simple base pairing mechanism between the single-stranded terminal sequence of the U1 snRNA molecule and the moderately conserved stretch of nucleotides at the 5′ss marking the exon-intron boundary [14]. That is why the presence of point mutations in this stretch can easily disturb U1 snRNA binding to the pre-mRNA region. Therefore, restoring the complementarity through engineered modification of the U1 snRNA represents a valuable therapeutic approach to correct the abnormal splicing process, which results from that disruption. In fact, this approach has been explored by a number of independent teams and applied to a significant number of mutations affecting the 5′ss. For this purpose, the normal 5′ end sequence of the U1 snRNA cloned in a vector is altered to a sequence complementary to the target mutated region, the so-called modified U1 snRNA vectors. These are designed to bind to the mutant pre-mRNA(s) by increasing their complementarity with the mutated 5′ss thus allowing the restoration of normal splicing [15]. Over the last decade(s), these sort of modified U1 snRNAs have been largely tested not only in cellular models of genetic diseases but also in vivo [16,17]. The primary goal of this review is to offer an overview of the role of modified U1 snRNAs and of their second-generation version, the exon-specific U1 snRNAs (ExSpeU1s) as therapeutic tools. Here we summarize recent studies that used this approach to correct the splicing process. In addition, we also comment on the importance of the pre-clinical validation and summarize the main obstacles that this innovative RNA therapy faces in order to be translated into the clinic.

## 2. The U1 snRNA Structure/U1 snRNP Complex and 5′ Splice-Site Features

**Figure 1:**
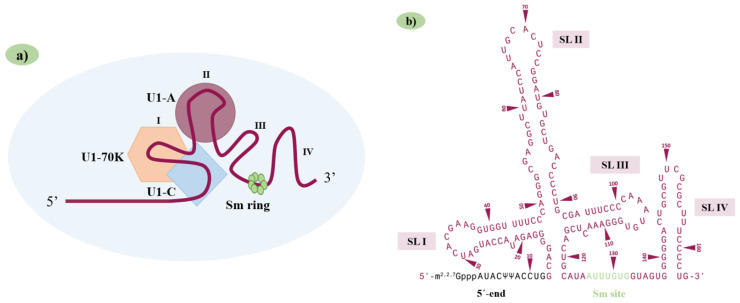
Illustrative figure of the U1 small nuclear ribonucleoprotein (U1 snRNP) complex and of the U1 small nuclear RNA (U1 snRNA) structure. (**a**) Summary of U1 snRNP topology. U1 snRNP (light blue) is composed of the U1 snRNA with 4 Stem-Loops (I-IV) (dark red line), 7 Sm proteins (green) and 3 U1 snRNP specific proteins: U1-70K (orange), U1-A (light burgundy) and U1-C (blue). (**b**) U1 snRNA sequence and structure. The m^2,2,7^G_ppp_ represents the snRNA-specific trimethyl cap structure. In U1 snRNA (164 nt), the 5′ end (black) binds to the 5′ splice-site and the downstream region contains the Stem-Loop (SL) I-III, followed by the Sm-site (green), and SL IV in the 3′end.

The U1 snRNP is one of the 5 snRNPs that constitute the spliceosome. This molecule is essential for the splicing process, and previous studies have shown that it is produced in larger quantities than any other snRNP, demonstrating its relevance. Mammalian U1 snRNP is comprised of a 164-nucleotide U1 snRNA, 7 Sm proteins (B or B’, D1, D2, D3, E, F, and G), which are shared with other snRNPs, and 3 U1-specific proteins (U1-A, U1-C, and U1-70K) (Figure 1a) [1,14,18].

In humans, one of the components of the U1 snRNP complex—the U1 snRNA, is encoded by a multigene family (known as *RNU1*) located on chromosome 1 (1p36) [19]. Imperfect copies of these U1 snRNA genes mapping to a separate location of chromosome 1 (1q12-21) were thought to be pseudogenes [20]. However, many of these ‘‘variant’’ (v)U1 snRNA genes produce fully processed transcripts, generating variant functional snRNAs. Studies in mice, frogs, and humans found expression changes of the canonical U1 and multiple U1 variant genes during cellular differentiation and development [21,22,23,24]. Furthermore, deletion of the chromosome region 1q12-21, involving all vU1 snRNA genes, causes severe neurological dysfunction reinforcing the notion that vU1 snRNAs may exert tissue-specific effects and be important for normal development. Therefore, the existence of a diverse population of vU1 snRNAs should not be ignored, as it can contribute to the fidelity and regulation of mRNA processing events in response to different environment stimuli, and have a significant impact on proteome diversity [25].

**Figure 2:**
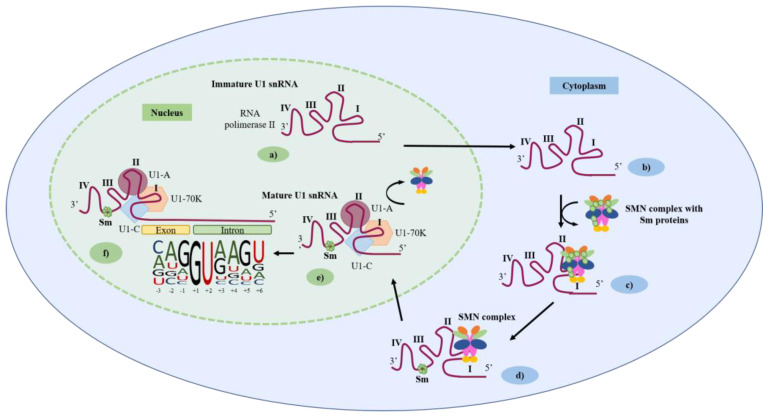
U1 small nuclear RNA (U1 snRNA) processing. The pre-U1 snRNA is transcribed in the nucleus by RNA polymerase II (**a**) and transported to the cytoplasm (**b**), where the formation of the Sm protein ring occurs (**c**,**d**). At this stage, however, the U1 snRNA is still immature. Then, the U1 snRNA returns to the nucleus, where the binding to the proteins U1-A, U1-70K and U1-C occurs and the U1 snRNA becomes mature (**e**). Finally, the mature U1 small nuclear ribonucleoprotein (U1 snRNP) initiates the spliceosome assembly through the binding to the pre-mRNA 5′ splice-site (9 nucleotides, CAG/GURAGU conserved between exons-introns) (**f**).

The U1 snRNA secondary structure consists of an unpaired 5′end, a four-way junction of 3 stem-loops (SL I-III) in a trefoil fold, a Sm site, and a fourth stem-loop at the 3′end (SL IV) (Figure 1b) [26]. This snRNA is transcribed in the nucleus by the RNA polymerase II (Figure 2a). Then, the pre-U1 snRNA moves to the cytoplasm (Figure 2b) where each of the 7 Sm proteins binds to a single base in the highly conserved Sm site (AUUUGUG) of the yet immature U1 snRNA, forming a ring structure in the following order: Sm-E, G, D3, B, D1, D2, and F. The formation of the ring structure is aided by the survival of the motor neuron (SMN) complex that is also involved in the assembly of snRNPs (Figure 2c,d). Following that, the U1 snRNA returns to the nucleus, where the U1-70K and the U1-A bind to SL I and II via specific motifs. The U1-C, on the other hand, does not bind to any U1 snRNA motif and is instead recruited to the U1 snRNP complex via protein:protein interaction with U1-70K and Sm-D3, ultimately resulting in the formation of the mature U1 snRNP (Figure 2e) [1,27]. This complex is now ready to start spliceosome assembly by binding to the 5′ss through base pairing between the 5′end of U1 snRNA (5′-m^2,2,7^G_ppp_AUACΨΨACCUG; Ψ—pseudouridine) and the moderately conserved stretch of nucleotides (CAG/GURAGU; R-purine) in the exon-intron junction (Figure 2f) [28]. However, not all base pairs at different 5′ss positions are equally important, and their contribution to splicing roughly correlates with their conservation. In eukaryotic organisms, in the 9 nucleotides consensus sequence (which can be expanded to include 11 base pairs), the most conserved 5′ss positions lie at the first two intronic positions (+1 and +2), which determine the 5′ss subtype. The GU subtype is present in 99% of the 5′ss, whereas the minor subtypes have a mismatch to U1 at either +1 or +2 and include the GC (0.9%) and the very rare AU 5′ss recognized by the spliceosome. The next most conserved 5′ss positions (>75% in humans) are -1G (the last exonic nucleotide) and +5G, which form strong G-C base pairing with the U1 snRNA, with three hydrogen bonds [18,29]. Consensus nucleotides −2A, +3A, +4A and +6U are also conserved but have a lesser although important contribution to 5′ss strength because their base pairing to U1 involves only the formation of two hydrogen bonds. The 5′ss positions +7 and +8 do not exhibit substantial conservation in humans, yet several lines of evidence indicate that these positions can base pair to U1 and contribute to 5′ss selection in mammals [29,30]. Thus, not all nucleotides located in the 5′ss region have an equal contribution to the splicing process: the more conserved, the greater their relevance (Figure 2f).

These different conservation levels in the 5′ss region turn it naturally diverse, with more than 9000 distinct sequences reported so far [29,31]. Relatively to the U1 snRNA sequence, approximately 40%, 22%, and 5% of the 5′ss include two, three, or four incompatibilities, respectively [32]. In fact, only about 5% of donor sites perfectly match the U1 snRNA consensus sequence, a value that could be substantially lower (0.85%) if only the most common canonical base pairing register is considered. However, the general helical U1 snRNA:5′ss duplex can accommodate alternative multiple non-canonical registers that include shifted base-pairing positions, bulged nucleotides on either the 5′ss or U1 snRNA strand, and asymmetric internal loops at different nucleotide positions, which contribute to increase complementarity and 5′ss selection. This reflects the huge flexibility and redundancy, that is allowed between the 5′ss-U1 snRNA base pairing to achieve efficient splicing at 5′ss [25,31,33]. In addition, this interaction is aided by several proteins such as U1-70K, U1-C, serine-arginine members and auxiliary elements, which may explain how the U1 snRNA is able to distinguish multiple pseudo-splice-sites [27,34,35].

Even though the U1 snRNP is known for its involvement in the splicing process, it does exert additional functions in the cell such as initiating transcription, protecting the mRNA from degradation, as well as protecting the pre-mRNA against premature polyadenylation [14,18,25].

## 3. The U1 snRNA Molecule as a Promising Therapeutic Tool for Splicing Correction

**Figure 3:**
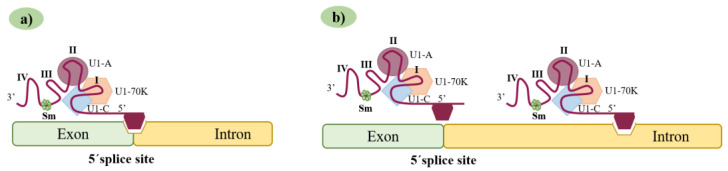
Schematic representation of U1 small nuclear RNA (U1 snRNA)-mediated therapeutic approaches for 5′splice-site (5′ss) mutations. (**a**) Mutation-adapted U1 snRNAs are designed to be complementary to the mutant 5’ss allowing a correct assembly of the spliceosome. (**b**) Exon-specific U1 snRNAs bind to a non-conserved intronic region promoting the use of the mutated 5’ss region through mechanisms not yet fully understood.

Together with the understanding of the constitutive splicing process and the perception of the crucial role U1 snRNAs play in it, came the realization that, by artificially generating and modifying U1 snRNAs, one could potentially correct aberrant splicing processes. To achieve this goal, two different generations of engineered U1 snRNAs are currently being explored: the modified U1 snRNAs and the ExSpeU1s (Figure 3). Here, we summarize the main results obtained in several studies where these two U1 generations were used to rescue aberrant splicing caused by different types of splicing mutations underlying different human genetic disorders (Table 1).

### 3.1. First Generation of Synthetic U1 snRNAs: The Modified U1 snRNAs

The use of modified U1 snRNA vectors that are similar to the endogenous U1 snRNA and have the ability to bind to the target pre-mRNA can restore splicing. For this, the 5′ end of U1 snRNA must be modified to a sequence that complements the mutated target sequence (Figure 3a). These modifications are made by site-directed mutagenesis on a plasmid that contains the U1 snRNA sequence, a promoter, and a termination sequence. The resulting modified U1 snRNA can then be transfected into cells, but it can also be inserted into adeno-associated viruses (AAVs) or lentiviruses and delivered both in vitro and in vivo [27,49,51].

In 1986, there was already evidence suggesting that the 5′ end of the U1 snRNA recognizes the 5′ss in mRNA precursors by complementary base pairing. In light of these facts, Zhuang and Weiner [67] tested for the first time whether modified U1 snRNAs can actually correct aberrant splicing patterns caused by mutations affecting the 5′ss. To do so, they checked whether the effect of point mutations in two alternative splice-sites of the adenovirus *E1A* gene was corrected by different modified U1 snRNAs. In normal conditions, the *E1A* pre-mRNA of the adenovirus is spliced into three mRNA species (13S, 12S, and 9S mRNAs) by the use of three alternative 5′ss. The presence of one point mutation at position +5 of the alternative 12S splice-site abolishes 12S mRNA synthesis. So, to try to correct the effect of that mutation, these authors constructed three modified U1 snRNAs using a plasmid vector and co-transfected it with the mutant *E1A* into HeLa cells. Remarkably, they verified that one of those U1 snRNAs was able to restore the 12S mRNA synthesis. In this same study, another point mutation was analyzed, in this case, it abolishes the production of the *E1A* 13S mRNA and is located at position +3 of the 13S 5′ss. Through the same protocol, three modified U1 snRNAs with three different matches to the 5′ss were designed. However, only a very weak mutation suppression was observed with one of those compensatory U1 snRNAs. These results further confirm that mutations, which reduce the match of a 5′ss to the U1 snRNA consensus sequence, usually impair splicing, and also suggest that this base pairing is necessary but not sufficient for the splicing of mRNA precursors. Most importantly, this discovery has unleashed numerous possibilities for the development of therapies for many diseases.

Subsequently, several studies in cellular models demonstrated that it was possible to correct disease-associated mutations in less conserved positions of the 5′ss. In fact, about a decade later, Baralle and colleagues [36] corrected a 5′ss variant causing the autosomal dominant disorder Neurofibromatosis type 1 (NF1). Their target was a substitution of a G for a C at the +5 position of the *NF1* intron 3 (c.288+5G>C). These authors used a minigene system containing the *NF1* exon 3 and its flanking intronic regions of both wild-type and mutant sequences. Transfection of the mutant minigene into Hep3B cells revealed that the G>C mutation in intron 3 drastically affects splicing leading to the skipping of exon 3, but when it was transfected together with an U1 snRNA fully complementary to the mutated site, the inclusion of the exon 3 was observed. To further prove this result, the authors also performed the co-transfection of the mutant minigene with a wild-type U1 snRNA vector and the recovery of the normal splicing process was not observed, highlighting that the correct splicing of exon 3 was due to the presence of the modified U1 snRNA molecule. Later, Pinotti and coworkers [38] also developed a U1 snRNA approach for the rescue of a 5′ss mutation causing Severe coagulation factor VII (FVII) deficiency. The target mutation in this study occurs at the +5 position of the *FVII* intron 7 [9726+5G>A (c.805+5G>A; according to current nomenclature)] and, results in the skipping of exon 7 leading to FVII deficiency. In an attempt to restore the normal splicing pattern, different complementary U1 snRNAs were designed and co-transfected with wild-type and mutant minigenes into COS-7 and Hep3B cell lines. The results showed that a totally complementary U1 snRNA improved the recognition of the correct 5′ss reducing exon skipping by 50% in both cell lines, an effect that showed to be dose-dependent. Afterward, the same authors [37] expanded this study, because although they had partially restored correct splicing using a totally modified U1 snRNA, the experimental settings did not allow for assessing its effect on protein levels and function, a key issue in the evaluation of the potential of a therapeutic approach. To solve this problem, the authors developed a full-length *FVII* splicing competent construct and co-transfected it together with the totally complementary U1 snRNA in COS-1 cells. As a result, a partial rescue of *FVII* splicing and FVII protein biosynthesis was observed. The result was dose-dependent, and it was the first time that a U1 snRNA-mediated rescue of splicing impaired by mutations at 5′ss resulted in an appreciable increase in functional protein.

Another example of the potential of these therapies came from the work developed by Tanner and co-workers [40] that used an adapted U1 snRNA for correcting the effect of a mutation causing Retinitis pigmentosa (RP). The target mutation (c.936G>A) involves the last nucleotide of exon 4 of the rhodopsin gene (*RHO*) and leads to two aberrant transcripts, one presenting the total skipping of exon 4 and the other showing a 4-base pair (bp) deletion at the end of exon 4 due to the activation of a cryptic splice-site. As in the previous studies, the authors started by addressing the therapeutic potential of modified U1 snRNAs using two minigenes, one containing the wild-type sequence and another harboring the mutant sequence that was transfected into COS-7 cells. Besides that, in order to assess plasmid expression in photoreceptor cells, these minigenes were also transfected into mouse retinal explants. Once again, a U1 adapted to the mutation led to increased levels of exon recognition and significantly reduced exon skipping. Nevertheless, activation of the cryptic splice-site was still detected. This may be explained by the fact that the target mutation is not only weakening the constitutive splice-site but is simultaneously activating the cryptic one. Another important study of the same group, targeting the same disease but focusing attention on another pathogenic mutation, was carried out by Glaus et al. [41]. These authors showed the efficacy of a fully complementary U1 snRNA to overcome the exon skipping caused by a mutation at +3 position in intron 10 (c.1245+3A>T) of the retinitis pigmentosa GTPase regulator (*RPGR*) gene, which origins an X-linked form of RP. Contrary to previous studies, the evaluation of the efficacy of the modified U1 snRNAs was performed in patient fibroblasts and four different U1 snRNAs were generated. Primary control and patient fibroblasts were then transduced with lentiviral vectors expressing the four U1 snRNA variants and the use of the U1 snRNA fully complementary to the mutation resulted in a partial correction of exon skipping. The treatment also revealed the use of cryptic splice-sites and the authors suggested that this might be related to the base change involved (A to T) that strongly disturbs splicing, since in the +3 position the most frequent bases are A or G, while T and C are much less frequent.

By the same time these pivotal studies were published, the use of homozygosity mapping in a large consanguineous family allowed to identify yet another disease-associated splice-site mutation: the c.479G>A variant in exon 5 of the *BBS1* gene, causing Bardet–Biedl syndrome. This mutation gives rise to three transcripts, one with the inclusion of intron 5 in addition to the constitutively spliced exons, one presenting the exon 5 skipping and unexpectedly, the correct spliced transcript was found although at reduced expression levels. Fully aware of the potential of modified U1 snRNAs to correct this sort of abnormal splicing, the researchers who identified this novel mutation also developed modified U1 snRNAs to increase their binding affinity for the mutated transcript. They also generated several minigenes that were co-transfected with four different modified U1 snRNAs in COS-7 cells. Furthermore, to better evaluate the efficacy of this approach, the patient’s fibroblasts carrying the c.479G>A variant were also transduced with lentiviruses containing the same adapted U1 snRNA sequences. The results revealed a significant increase in correctly spliced *BBS1* transcripts and, simultaneously, a reduction in transcripts lacking exon 5. Of note, the transcript with intron 5 retention was still observed [42].

Overall, there are many other genetic disorders for which the use of modified U1 snRNAs can be attempted for therapeutic purposes. For example, splicing defects have a significant role in Propionyl-CoA carboxylase deficiency, with the majority of mutations being located either in the 3′ss or the 5′ss. Thus, attempting splicing modulation seemed to be a promising approach to address some of those mutations, namely the c.1209+3A>G mutation, which causes the skipping of the exon 13 of the *PCCA* gene that encodes the protein, which is deficient in this syndrome. So, in 2011, Sánchez–Alcudia and co-workers [43] investigated the potential of modified U1 snRNAs to correct its effect. Initial studies were performed in Hep3B cells with minigenes, but the corresponding wild-type construct failed to recapitulate accurately the transcriptional profile observed in fibroblasts probably due to the lack of the whole genomic sequence context. Therefore, patients’ fibroblasts homozygous for the nucleotide change were used as a cellular model for transfection with U1 constructs with different compensatory modifications. Using this strategy, the authors observed that when three of the four adapted U1 snRNA vectors were transfected, a reversion of the splicing defect could be detected, but only with the fully modified U1 snRNA the levels of the normal spliced band with exon 13 reached ~100%. However, the correction of the splicing defect did not result in a significant increase in enzymatic activity, highlighting the existence of other factors still unknown that can affect the successful application of these therapies.

Another curious example comes from the work of Scalet and colleagues [44] that studied the splicing mutation c.1062+5G>A in the *FAH* gene, which causes Tyrosinemia type 1. This mutation involves the *FAH* exon 12 5′ss and prevents its usage, even when forced by a modified U1 snRNA designed to increase the mutant 5′ss recognition. Noticeably, these authors verified that in one patient the presence of a new somatic change involving the penultimate position of the exon 12 (c.1061C>A) in linkage with the c.1062+5G>A mutation, partially rescues the defective 5′ss. Interestingly, this combined genetic condition strongly favored the rescue by a modified U1 snRNA that promoted the inclusion of exon 12 with a robust synthesis of correct transcripts that reached up to 60%. Therefore, this study clearly demonstrates the complexity associated with the development of U1 snRNA therapies.

Overall, the studies reviewed so far have addressed the capacity of modified U1 snRNAs to restore the splicing process in the presence of mutations affecting less conserved regions of the 5′ss. However, there are also works reporting the correction of mutations affecting the more conserved positions of the 5′ss that is, those located at +1 or +2 sites. To the best of our knowledge, though, there are only three reports on the successful correction of this type of mutation: one for the Fanconi anemia C (FANCC) [45], other for the rare Lysosomal Storage Disease Mucopolysaccharidosis type IIIC (MPS IIIC) [11], and the third one for Shwachman–Diamond Syndrome [46].

In the first case, Hartmann and co-workers [45], showed a partial correction of a +1 mutation (c.165+1G>T) in the intron 2 of the *FANCC* gene, which is associated with a milder form of FANCC. This work was the first successful correction of a +1 mutation, which converts the highly conserved GT dinucleotide within the 5′ss of *FANCC* exon 2 to a TT dinucleotide resulting in aberrant splicing. The majority of the observed transcripts (73%) were non-functional due to exon skipping or the usage of cryptic splice donor sites. Unexpectedly, however, the presence of the mutant TT 5′ss also enabled a correct recognition of the normal exon-intron boundaries, leading to reduced amounts of normal transcripts (27%). Briefly, the authors used two different cellular systems, HeLa cells with a splicing reporter construct carrying the target mutation and patient-derived fibroblasts and demonstrated that transfection with two different TT-adapted U1 snRNA expression plasmids facilitated correct exon 2 recognition, thereby increasing the fraction of *FANCC* exon 2 in-frame spliced pre-mRNA. Finally, the use of lentiviral vectors as a delivery system to introduce the mutation-adapted U1 snRNAs into primary FANCC patient fibroblasts allowed the rescue of the pathological phenotype of these cells. Interestingly, the level of functional restoration of transduced cells differed between the two U1 snRNAs that were specifically adapted for the mutant *FANCC* exon 2 5′ss. The fully complementary U1 snRNA allowed to achieve a good correction level while the adapted U1 snRNA with only a single compensatory mutation was less efficient.

In the second case, the use of modified U1 snRNAs to correct *HGSNAT* gene splicing mutations was also attempted by Matos and collaborators [11] as an alternative therapeutic strategy for MPS IIIC. These authors tried to overcome the effect of three *HGSNAT* 5′ss mutations: c.234+1G>A, c.633+1G>A and c.1542+4dupA. The first two affect the position +1 of the donor splice-site causing the skipping of the corresponding exons. The c.234+1G>A mutation, leads to the skipping of exon 2 and the c.633+1G>A causes the skipping of exon 6. Regarding the c.1542+4dupA, this affects the position +4 of the 5′ss of exon 15 and origins two transcripts: one with the skipping of exon 15 and the other containing the exon 15 plus the five first nucleotides of intron 15 due to the presence of a cryptic splice-site at the beginning of this intron.

As many other authors did in the previously reported studies, a first optimization approach where the effect of different modified U1 snRNAs on the splicing process was tested on minigenes bearing each mutation, but no correction of the aberrant splicing was observed. In fact, even though the skipping of the respective exon was corrected, the transcript generated after treatment was still aberrant due to the use of cryptic “gt” donor sites situated at the intronic positions +5 and +6 (+1 mutations) or +6 and +7 (+4 dupA mutation) that was promoted, partially or completely, by some adapted U1 snRNAs. Still, taking into account that minigenes do not entirely reproduce the human-specific genomic context, the different U1 variants were also tested directly into patients’ fibroblasts. In the case of the c.633+1G>A and c.1542+4dupA mutations, the correct endogenously splicing process was not recovered after the overexpression of the different modified U1 snRNAs. Remarkably, however, for the c.234+1G>A mutation, which was present in homozygosity in two patients, the overexpression of a modified U1 that completely matches the mutated 5′ss, did promote a partial correction (almost 50%) of the splicing process, together with the use of the cryptic “gt” site at intronic positions +5 and +6. This was an unexpected and surprisingly positive result since the majority of the mutations localized in the high conserved nucleotide positions +1 and +2 of the donor splice-site that were submitted to correction with modified U1 snRNAs were unable to be rescued.

Another example in which positive results were observed came from a recent study [46] involving a very common 5′ss variant that is present in more than 90% [68,69] of worldwide patients with Shwachman–Diamond syndrome, one of the most common inherited bone marrow failure syndromes. The c.258+2T>C variant at the 5′ss of *SBDS* gene exon 2 is associated with aberrant pre-mRNA splicing leading to exon 2 skipping but trace levels of correct transcripts (2%) are also detected by qPCR, which account for residual protein expression and explain the survival of affected patients [46].

Recently, the same team that assessed exon 2 inclusion efficiency in c.258+2T>C lymphoblastoid cells, also tested, in vitro, different therapeutic approaches for this mutation, which included the test of a panel of engineered U1 snRNAs either modified or exon-specific (see Section 3.2 for information about this kind of U1 snRNAs). *SBDS* minigenes (wild-type or mutated) were co-transfected with one modified U1 snRNA or three different ExSpeU1 variants in HEK293T cells. All modified U1 snRNAs strongly promoted the use of a proximal cryptic exonic 5′ss and two ExSpeU1s also promoted the use of an exonic cryptic 3′ss. However, the modified U1 led also to a slight increase in the relative proportion of the corrected transcripts meaning that forcing the definition of the defective exon by using a compensatory U1 snRNA, albeit with low efficacy, did promote the synthesis of correct transcripts [46].

Overall, the promising results obtained in vitro for the majority of disease-causing-variants, whose effect over splicing was total or partially corrected through the action of modified U1 snRNAs, prompted additional studies to assess the feasibility of this approach in vivo. Depending on the target mutation and on the phenotypic presentation of the associated disorder, different administration methods have been attempted, leading to either transient or continued expression of the therapeutic U1 snRNAs. In vivo, the administration of modified U1 snRNA vectors can be performed via hydrodynamic injection, which allows their transitory expression. To obtain continued expression of the modified U1 snRNAs, however, it is necessary to use viral vectors. Among these, the most used in in vivo studies are the AAVs [39,70]. Indeed, AAVs have many advantages, such as a wide biodistribution in tissues, being accepted by the animals, their capsid has low immunogenicity compared to other viruses and, finally, they have the necessary packaging capacity to incorporate the U1 snRNA sequence [71].

One of the first in vivo studies using a modified U1 snRNA was performed by Balestra et al. [39]. These authors performed both transient and stable expression of constructs with a splicing mutation that causes FVII deficiency in humans, which they intended to correct. For the transient expression, they used plasmid constructs, while for the stable one, they used AAV vectors. Briefly, to obtain the desired correction, in the transient expression experiments, the plasmids carrying the human *FVII* splicing mutation and the modified U1 snRNA adapted to the mutation at position +5 of intron 7 [859+5G>A (c.805+5G>A; according to the current nomenclature)] were simultaneously administered by hydrodynamic injection. For the continued expression experiments, on the other hand, two AAV vectors of different serotypes, one containing the human *FVII* pre-mRNA and the other the adapted U1 snRNA were sequentially administered by intravenous (IV) injection. Remarkably, the authors demonstrated that, regardless of the expression method, the mutation-adapted U1 snRNA was able to rescue *FVII* gene splicing and improve clotting in C57BL/6 mice.

Later, Lee and co-workers also addressed the therapeutic potential of modified U1 snRNAs in vivo [16]. Having demonstrated in N2A cells that a totally modified U1 snRNA was able to improve the splicing of a minigene bearing the IVS6+4A>T (c.714+4A>T) mutation of the *AADC* gene, they checked whether a similar effect was observed on mouse sequences from an artificial splicing construct using the same cell model. Since they succeeded, they moved on to in vivo studies, showing a therapeutic effect in knock-in mice with Aromatic L-Amino Acid Decarboxylase (AADC) deficiency after intracerebroventricular (ICV) injection of the AAV9 carrying the fully modified U1 snRNA. The treatment had promising results with an improvement in the survival and in the brain dopamine and serotonin levels of the mice.

In another study, Balestra and colleagues [13] used a mouse model (FAH5961SB) for the Fumarylacetoacetate hydrolase (FAH) deficiency (also known as Tyrosinemia type 1) and demonstrated that a compensatory U1 snRNA expressed in AAV, and administered via the lateral tail vein was able to partially restore the normal splicing disrupted by a mutation at the last nucleotide of the mouse *Fah* gene exon 8 (c.706G>A). Importantly, there was no evidence of toxicity in this trial, even though the same was not true for the first in vivo study of Balestra et al. [39] for FVII coagulation deficiency, where the authors did observe some hepatotoxicity in mice expressing the human *F7* c.805+5G>A mutant after modified U1 snRNA overexpression, which was attributed to off-target effects.

### 3.2. A New Generation of Synthetic U1 snRNAs: The Exon-Specific U1 snRNAs

In addition to the modified U1 snRNA vectors whose modifications are based on the target 5′ss sequence, there is yet another U1 snRNA-based strategy being developed and applied. These new molecules are designated exon-specific U1 snRNAs (ExSpeU1s) (Figure 3b) and have been developed to increase the specificity and the ability to include the target exon while significantly reducing the possibility of occurring off-target effects [18,48]. These ExSpeU1s are designed to recognize very poorly conserved regions of a given intron, downstream of the 5′ss of a target exon [72]. This means they are specific to a certain exon, and that is why they are given the designation ExSpeU1s. Unlike antisense oligonucleotides (ASOs), these molecules do not block intronic elements, but promote spliceosome activation through the recruitment of splicing factors improving exon definition [52]. Thus, ExSpeU1s have a broader range of therapeutic applications when compared to their previously referred counterparts. Theoretically, ExSpeU1s can be used to correct exon skipping caused by mutations in either the 5′ss, 3′ss, polypyrimidine tract or exonic regulatory elements. In fact, these molecules proved to be quite efficient both in vitro and in vivo, and a significant number of studies developed ExSpeU1s to correct different types of splicing mutations associated with defective exon definition in several human disorders [18].

The overall potential of this approach was assessed for the first time back in 2012, in a work from Franco Pagani’s lab by Alanis and collaborators [48]. This team used ExSpeU1s to rescue splicing mutations in three different genetic diseases. They started by characterizing natural mutations in *F9* exon 5, *CFTR* exon 12 and *SMN2* exon 7 associated with exon skipping in Hemophilia B, Cystic fibrosis (CF), and Spinal muscular atrophy (SMA), respectively and then tried its therapeutic splicing rescue by using different ExSpeU1s. For each gene system (*F9* exon 5, *CFTR* exon 12 and *SMN2* exon 7), they identified an ExSpeU1 able to rescue splicing impaired by different types of mutations. More specifically, they showed that the ExSpeU1-mediated correction of different *F9* exon 5 mutations in the 5′ss (3 synonymous mutations in position −2: −2C, −2G and −2T; see Table 1) and in the polypyrimidine tract (2 intronic variants: −8G and −9G; see Table 1), results in complete restoration of secreted functional factor IX levels. However, for 5′ss mutations at the positions −1T, +1A and +2C (see Table 1), the ExSpeU1 did not correct the splicing defect. Regarding Cystic fibrosis, the 5′ss mutations in *CFTR* exon 12 showed a variable response to ExSpeU1, in this case, the rescue efficiency was optimal (~85%) for the +3G, intermediate (~50%) for the −1A, +3C and +5A and low (~20%) for the −1T (see mutations in Table 1). Furthermore, this ExSpeU1 allowed the almost complete splicing correction of two *CFTR* exon 12 mutations (see Table 1). Similarly, for SMA it was demonstrated in minigene assays and in the chromosomal context of normal cells (HEK293) that ExSpeU1s also improved *SMN2* exon 7 splicing. The SMA is a genetic neuromuscular disease caused by loss-of-function mutations in the *SMN1* gene, but humans have a paralogue gene called *SMN2*. One of the few nucleotide changes between *SMN1* and *SMN2* genes is a silent mutation within exon 7 (c.840C>T) that makes 80–90% of the transcripts derived from the *SMN2* gene skip exon 7. Thus, the modulation of *SMN2* exon 7 splicing has been on the basis of several therapeutic attempts for SMA. In fact, another study performed also by Pagani’s team [53], showed that the same ExSpeU1s developed by Alanis et al. [48] for SMA significantly corrected endogenous *SMN2* exon 7 splicing and restored the levels of the resulting SMN protein in two cellular models (HEK293 Flp-In cells and lentiviral-infected SMA type I fibroblasts). Interestingly, the results demonstrate that the ExSpeU1 snRNA molecule not only corrects splicing but also increases mRNA levels and stabilizes the *SMN* pre-mRNA intermediate. This effect resulted in higher levels of *SMN* mRNAs when compared to those achieved in the treatment with an ASO that targets corresponding intronic sequences. The outcome of pre-mRNA processing can be explained because while the ASO acts as an antisense molecule masking an intronic splicing silencer (ISS), the ExSpeU1s recruits splicing factors on the defective upstream exon and decreases the mutation effect.

To evaluate the activity of ExSpeU1 in vivo, the authors also tested it in newborn mice carrying the *SMN2* transgene through AAV-mediated delivery. The RT-PCR showed an increase in exon 7 inclusion in the brain, heart, liver, kidney, and skeletal muscle. This result demonstrates that ExSpeU1 can be efficiently expressed in vivo in different mouse tissues and exert an effect on its natural human *SMN2* target [53].

In another study, also for SMA and conducted by the same lab [51], a transgenic mouse expressing an ExSpeU1 was bred and crossed with a severe SMA mouse model to assess the therapeutic potential of this specific sort of U1 snRNAs for erroneous splicing correction. The results showed an increase in the inclusion of *SMN2* exon 7, production of SMN protein, and a significant improvement in phenotype and survival. For the first time, a specific modification of a core spliceosomal component proved to be safe and effective in a severe SMA mouse model as a novel therapeutic strategy for this disorder. However, this was not the only contribution that came from this study when it comes to the overall ExSpeU1 state of the art. In fact, besides the in vivo analysis, the authors have also made an attempt to evaluate in vitro the structural requirements of these ExSpeU1 particles and showed that the U1 snRNP 70K protein and the stem-loop IV elements of the U1 snRNA mediate most of the splicing rescue activity through improvement of exon and intron definition.

Subsequently, this study was expanded and the team tested the viral delivery of the *SMN2* ExSpeU1 encoded by the adeno-associated virus AAV9. Overall, the virus increased *SMN2* exon 7 inclusion and SMN protein levels and rescued the phenotype of mild and severe SMA mice. Furthermore, the treatment improved the neuromuscular function and increased the life span from 10 to 219 days in severely affected mice. Quite remarkably, ExSpeU1 expression persisted for 1 month and was effective at around one five-hundredth of the concentration of the endogenous U1 snRNA. Moreover, when the same authors analyzed, through RNA-sequencing (RNA-seq), a human cellular model overexpressing the ExSpeU1 more than 100-fold above the therapeutic level, no damaging effects were observed, i.e., it did not significantly alter global gene expression or splicing. Overall, the high efficacy in two SMA animal models, the lack of off-target effects on the human transcriptome and the long-lasting effect observed by these authors, further support the idea that ExSpeU1s delivered by AAV may be a valid therapeutic tool for splicing-related pathologies [52].

Overall, the pool of knowledge that has been accumulating over the last decade on the therapeutic potential of ExSpeU1 goes far beyond SMA. Other important clues on the applicability and translatability of the ExSpeU1 approach in rescuing exon skipping defects, came precisely from the study of yet another of the three original disorders addressed by Alanis and co-workers [48] in their proof of principle paper: Cystic fibrosis. In fact, when Donegà and colleagues [50] attempted an ExSpeU1-based approach to rescue a total of 10 different CF-causing splicing mutations, their overall results were extremely positive. The authors chose 10 relatively frequent splicing mutations that cause the skipping of 5 different exons (5, 10, 13, 16 and 18; see Table 1). Splicing mutations 711+3A>C/G (c.579+3A>C/G) and 711+5G>A (c.579+5G>A) are located in the 5′ss consensus of exon 5; 1863C>T (c.1731C>T) and 1898+3A>G (c.1766+3A>G) in an exonic regulatory element and in the 5′ss consensus of exon 13, respectively; 2789+5G>A (c.2657+5G>A) and 3120G>A (c.2988G>A) are located at the 5′ss consensus of exon 16 and 18, respectively, whereas TG13T3, TG13T5, TG12T5 are variants at the polypyrimidine tract of exon 10. Through a somehow classical, yet elegant, minigene-based splicing assay, the authors proved that all those mutations could be efficiently corrected in vitro by a panel of ExSpeU1s engineered to bind to intronic sequences downstream of each defective exon. Most importantly, they also showed that the ExSpeU1-mediated splicing correction at the RNA level did correlate with a recovery of the full-length CFTR protein for some of those variants. Additionally, from the panel of mutations analyzed in this study, those involving exon 13 skipping were shown to be less prone to an efficient ExSpeU1-mediated correction. Therefore, the authors further analyzed exon 13 splicing, focusing attention on intronic regulatory elements within the ExSpeU1 binding region and performing a detailed mechanistic analysis, which eventually disclosed why exon 13 was the most challenging to rescue. In summary, this was the first time that an exon rescue strategy was shown to work with such efficiency in a single gene on several exon-skipping mutations and different exons [50], but soon evidence started to gather on the overall potential of ExSpeU1s as therapeutic molecules, with a number of other diseases and mutations adding up to the targets catalog. For example, the effect of different ExSpeU1s and modified U1 snRNAs was evaluated for mutations in the *ATP8B1* gene. ATP8B1 deficiency is a severe autosomal recessive liver disease characterized by a continuous phenotypical spectrum from intermittent to progressive Familial intrahepatic cholestasis. The differences in disease severity may be caused by variations in the structure and/or function of the ATP8B1 protein resulting from the type of mutation. As in other trials described throughout this review, Van der Woerd and co-workers [10] also used a minigene system to evaluate splicing effects caused by variants at exon-intron boundaries, as well as to test the ability of complementary modified U1 snRNAs and ExSpeU1s to rescue aberrant splicing. The first ones have been shown to completely restore splicing for two 5′ss *ATP8B1* mutations (c.279G>A; c.625_627+5delinsACAGTAAT) and to improve the normal splicing process for the first time of one mutation located at the position −3 of the 3′ss (c.2932-3C>A). However, for the c.2418+5G>A splicing correction was not achieved. Furthermore, the authors evaluated five ExSpeU1s that bind at different intronic positions downstream of *ATP8B1* exon 7 for mutation c.625_627+5delinsACAGTAAT. Although very effective in correcting exon skipping, the ExSpeU1 treatment also resulted in partial retention of the short downstream intron.

In another study, Balestra and colleagues [47] also evaluated the capability of modified U1 snRNA and ExSpeU1 variants to correct mutations affecting +1 and +5 nucleotides at the 5′ss, which are predicted to cause *CDKL5* exon skipping. Mutations in the *CDKL5* gene lead to the CDKL5-deficiency disorder, a rare neurological condition characterized by the onset of seizures in the first weeks of life and severe intellectual disability. In this study, the authors used the two variants of U1 snRNA, the modified U1 snRNA, and the second-generation ExSpeU1. However, both failed to restore exon definition in the presence of nucleotide changes occurring at position +1 (c.99+1G>T; c.463+1G>A; c.744+1G>C) in multiple exons of *CDKL5* gene. On the other hand, both generations of U1 snRNA variants completely rescued exon definition in the presence of the mutations c.99+5G>A and c.2376+5G>A located in the exon 3 and 16 contexts, respectively. Nevertheless, since the modified U1 snRNA improved *CDKL5* splicing in a dose-dependent manner and the ExSpeU1 completely restored proper exon definition at the lowest dose tested, only the ExSpeU1 was considered for protein assays. The results showed that this molecule fully restores CDKL5 protein synthesis, subcellular distribution and kinase activity further supporting its therapeutic potential.

Adding up to the catalog of severe genetic diseases, which may be caused by splicing mutations potentially amenable to U1 snRNA-based therapies, are a number of hereditary bleeding disorders, such as Hemophilias A and B. The first of those disorders was precisely Hemophilia B. In fact, back in 2016, Pagani’s Lab [12] addressed a series of exonic splicing mutations (synonymous, missense and nonsense) in exon 5 of the *F9* (*FIX*) gene that causes Coagulation factor IX disease, also known as Hemophilia B. These authors studied the impact of those mutations on mRNA splicing and/or protein biology as well as their possible therapeutic rescue by the ExSpeU1 approach. Through expression studies with splicing-cassette minigenes and full-length protein constructs, the authors identified two synonymous substitutions (p.V107V; p.R116R) causing exon skipping and two missense mutations (p.A118V; p.Q121H) with partial effects on splicing and coagulation factor IX (FIX) secretion, but in which the spliced-corrected proteins maintain normal FIX coagulant activity as potential targets for a splicing-switching therapy. Furthermore, they tested different ExSpeU1 molecules binding intronic sequences downstream of *FIX* exon 5 and found an active ExSpeU1 (U1-fix9), which completely rescued aberrant exon skipping caused by different exonic mutations, through an SRSF2-mediated mechanism. Interestingly, the same team had already shown in previous work [48] that this ExSpeU1 recovered exon skipping caused by 5 mutations located at the polypyrimidine tract or at the 5′ss of *FIX* exon 5. So, together both studies identified different splicing mutations causing Hemophilia B, which may likely have a therapeutic benefit from splicing correction by a unique ExSpeU1 molecule.

In general, the evaluation of the safety and efficacy of this kind of therapy is limited by the availability of cellular and animal models specific to a given mutation. Remarkably however, Barbon and co-workers [49] were actually able to assess the efficacy of the ExSpeU1 fix9 (U1-fix9) reported above in cellular and mouse models that were generated using a specific transposon system to integrate splicing-competent human *FIX* minigenes either wild-type or carrying the c.519A>C splicing mutation in *FIX* exon 5 into the genome of HEK293 cells and C57BL/6 mice. Both models were then used to test, in a genomic DNA context, the efficacy of the U1-fix9, delivered with an AAV8 vector to overcome the effect of the splicing variant found in patients with Hemophilia B. The results showed the correction of the splicing pattern of human *FIX* mRNA, which translated into an increase in FIX protein expression levels.

Still on Hemophilia-related pathologies a very interesting study was conducted by Balestra et al. [54] that challenged the efficacy of a U1 snRNA-mediated strategy for splicing mutations occurring at the 3′ss, 5′ss or within exon 5 of the *F8* (*FVIII*) gene (see Table 1) and associated with different severity degrees of Coagulation factor VIII (FVIII) deficiency (Hemophilia A). The *F8* exon 5 is a singular model in which the original 5′ss has several cryptic 5′ss nearby, a scenario that becomes even more complex in the presence of nucleotide alterations at the 5’ss. So, taking this model and using minigenes, the authors demonstrated for the first time that a unique ExSpeU1 targeting an intronic region downstream of exon 5 was able to redirect the use of the canonical 5’ss (~80%) in the presence of several mutations located at 3′ss, 5′ss or within the exon. Once again, a more detailed investigation of the rescued transcripts from +1 and +2 intronic variants showed that the ExSpeU1 promoted the use of adjacent cryptic 5′ss, leading to frameshifted transcript forms, which eventually compromised the correction attempt.

A similar approach was later developed by the same team for the correction of several *F8* gene mutations affecting the splicing of exon 19 (see Table 1) [55]. This exon was chosen as a model because it is poorly defined, which points towards the existence of functional regulatory elements promoting its proper inclusion. An extended panel of disease-causing exon 19 variants was challenged by a correction approach with engineered U1 snRNAs. First, the authors used a *F8* exon 19 minigene and tested in vitro the effect of one complementary U1 snRNA and three ExSpeU1s in the rescue of the c.6115+4A>G variant, which was previously associated with total exon skipping. The most efficient, which was one of the ExSpeU1s, was then used to correct the other exon 19 splicing affecting variants and was very efficient in the rescue of all exonic variants as well as of three intronic variants (exon inclusion ranging from +18% to +68%). Only for three mutations, c.6115+1G>A, +2T>C and +5G>A, the use of the ExSpeU1 led to the activation of a cryptic 5′ss, which resulted in partial intron 19 retention. However, the co-delivery of the ExSpeU1 with a modified U6 snRNA partially rescued (from 0 to 3%) the effect of one of those variants (c.6115+5G>A; see Section 3.3 for combined therapeutic strategies).

Overall, these studies demonstrate that a single ExSpeU1 can efficiently rescue different mutations involving the same exon adding up to the pool of knowledge on the plasticity of single ExSpeU1s and making these molecules particularly attractive for the correction of rare genetic variants. In fact, the development of this kind of approach has a great therapeutic potential for rare diseases, particularly for splicing mutations that are relatively frequent among patients with a specific rare disease. That is the case of the work conducted by Dal Mas et al. [56] in which an ExSpeU1 approach was tested to correct the splicing defect caused by a frequent synonymous variant (c.891C>T) in the *SPINK5* gene, which resulted in Netherton syndrome (NS), a rare severe autosomal-recessive genodermatosis. This variant is located in the position +9 of exon 11 and induces its skipping. Briefly, different ExSpeU1s targeting the intronic region downstream of the exon 11 constitutive 5′ss were tested through minigene splicing assays and rescued with different efficacies the skipping of the exon 11. Then, the most active ExSpeU1 was tested in keratinocytes from a NS patient bearing the target variant. Its lentiviral-mediated transduction allowed to recover the correct *SPINK5* transcript and also the corresponding functional protein.

Similar approaches have been attempted for different diseases over the last decade, even though their results have not always been as promising as the previously reported. For example, virtually at the same time the previously referred ExSpeU1 proved to be efficient both at mRNA and protein level for that particular NS-causing variant, other ExSpeU1 failed to correct the effect of yet another 5′ss mutation (c.790C>T) in a patient with Fanconi anemia [57].

In another work, Pagani’s team [58] tested the therapeutic effect of ExSpeU1s in Familial dysautonomia (FD), a rare neurodegenerative genetic disease with no treatment, caused in more than 99% of the patients by a homozygous intronic point mutation (c.2204+6T>C) in the *ELP1* gene, which causes different degrees of exon 20 skipping in various tissues. In a first approach, the team tested eleven ExSpeU1s binding different regions of intron 20 through co-transfection with a mutant T>C minigene in neuronal SH-SY5Y cells and found that seven of the ExSpeU1s allowed a significant recovery of the splicing defect. Then, to better understand exon 20 splicing inhibition and to select the best U1 molecule to test both in the patient’s cells and in mice, different functional splicing assays were performed, and the results showed that two ExSpeU1s were the most effective on splicing recovery, being able to overcome the negative effect of different inhibitory splicing factors. Subsequently, to evaluate the effect of ExSpeU1 in FD patient’s fibroblasts, its lentivirus-mediated expression was performed, and the results showed a huge increase in the percentage of exon 20 inclusion consistent with a recovery of ~80% at the protein level. Next, the therapeutic potential of the ExSpeU1s was assessed in a transgenic mouse model that carries the *ELP1* splicing mutation and has the same tissue-specific mis-splicing observed in FD patients, but which does not exhibit any symptoms. The intraperitoneal (IP) delivery of ExSpeU1s-AAV9 particles successfully increased the production of the human *ELP1* full-length transcript and protein in several mouse tissues, demonstrating that the ExSpeU1 approach would be a potential therapeutic option for FD.

Later, in another study, to understand if the *ELP1* exon 20 splicing rescues achieved with the specific AAV9-ExSpeU1 in the FD asymptomatic mouse model is translated in an amelioration of the disease phenotype and life span, the same group tested this modified U1 molecule in a phenotypic humanized mouse model that exhibits the major disease symptoms and reproduced the human splicing defect. The postnatal systemic and ICV treatment with the AAV9-ExSpeU1 in these mice allowed an effective correction of the *ELP1* splicing and increased the production of a functional protein in several tissues. Furthermore, the treatment rescued FD mice’s motor, cardiac, and renal functions, and reduced animal mortality in the first month of life. Then, to evaluate possible off-target effects caused by the presence of the ExSpeU1, the authors did RNA-seq analyses in human cells overexpressing the specific U1 and in the dorsal root ganglia of treated mice, and did not observe significant changes in gene expression and alternative splicing, a result that together with the potentially long-lasting splicing correction provided by the systemic treatment of AAV9-ExSpeU1 make this a promising therapeutic strategy for FD [59].

Remarkably, the engineered U1 snRNA vectors can also be tested in N-of-1 treatment approaches and be developed for variants that are present in a single patient/family or only in a few patients. One such example is a very recently developed study by Jüschke et al. [60] focused on patients with Autosomal dominant optic atrophy (ADOA), an ocular disorder frequently caused by mutations in the *OPA1* gene, and in which haploinsufficiency is the main genetic pathomechanism. They identified a family with ADOA harboring a novel 5′ss mutation, c.1065+5G>A, in intron 10 that causes exon 10 skipping and leads to a reduction of ~50% of OPA1 protein expression. Similarly, to what Balestra et al. had executed in 2019 for CDKL5-deficiency disorder, these authors also tested the effect of the two different generations of synthetical U1 snRNAs. Briefly, in order to correct the mutation-induced splicing defect, the team designed five different modified U1s: a U1 variant with full complementarity to the mutated 5′ss of exon 10 and four ExSpeU1s adapted to different regions downstream of the 5′ss in intron 10. Their lentiviral transduction in patient-derived fibroblasts showed that only the ExSpeU1 directed to the position +18 in intron 10 was able to significantly reduce the transcripts with exon 10 skipping and simultaneously increase the expression level of normal transcripts in a dose-dependent manner. Furthermore, a tendency towards increased OPA1 protein expression was associated with the treatment with this ExSpeU1. So, the authors hypothesized that the increased amount of correctly spliced *OPA1* transcripts may be sufficient to surpass the haploinsufficiency and lead to an amelioration of the phenotype and to a slower disease progression. To the best of our knowledge, there is not any published data on the in vivo effect of this ExSpeU1 yet. Still, the in vitro results shared so far seem quite promising.

As in the previous study, Martinez–Pizarro and collaborators [61] also studied two very rare intronic variants (c.1199+17G>A and c.1199+20G>C) located in intron 11 of the *PAH* gene, which cause exon 11 skipping and origin one of the most common inherited disorders of amino acid metabolism, Phenylketonuria. Again, several ExSpeU1s were tested as therapeutic tools for the mentioned variants involving intronic positions downstream of the 5′ss, also allowing us to gain more insights into the cellular functions of the natural U1 snRNP. Through different functional splicing studies, the authors identified a novel region in intron 11 that functions as an intronic splicing enhancer (ISE) for the exon 11, which has a weak 3′ss and a suboptimal 5′ss. This ISE has a binding motif for U1 snRNA and RNA affinity assays showed that U1-70K binds to this region in the wild-type sequence. However, this binding is abolished in the presence of the two intronic variants which reduce the complementarity to U1 snRNA. Overall, this shows that the splicing regulatory element in intron 11 is relevant for the regulation of correct splicing at the canonical 5′ss. Further evidence of the role of this ISE for exon 11 inclusion was obtained by the results of splicing therapeutic approaches using different ExSpeU1s in co-transfection with mutant minigenes. Only the ExSpeU1s which were totally complementary to the +17G>A or +20G>C mutations did increase exon 11 inclusion. This demonstrates that in this context the binding of each specific U1 to the intronic sites possibly compensates for the defect by recruiting the U1-70K protein (identified as a mediator in the splicing rescue by ExSpeU1s, see [51]), thus allowing to restore the interactions that define exon 11 and achieve a therapeutic effect. Of note, this work extended the knowledge about the mechanism of action of the natural U1 snRNPs showing that in some contexts it can act as a splicing stimulator particularly when it binds to an intronic region downstream of the natural 5′ss.

In summary, the ability of different engineered U1 snRNAs to rescue splicing has been proven in both cellular and animal models of different human genetic disorders. However, the existence of species-specific splicing profiles can constitute a challenge to the development and validation of these therapies. This is elegantly presented in two recent studies developed by the same group [62,63], both targeting a splicing variant (c.386G>A) in the *OTC* gene, the gene mutated in Ornithine transcarbamylase deficiency (OTCD). The presence of the c.386G>A variant in the last base of exon 4 leads to aberrant splicing but with a substantially different impact between humans and mice. In mice, this variant causes exon 4 skipping and the use of a cryptic intronic 5′ss at position +49, but the correct transcript is still present in appreciable amounts. In turn, in humans, the same variant also leads to exon 4 skipping but that is the major similarity between both systems. Indeed, in humans, that same variant leads to the use of a cryptic 5′ss at the +5 position and only trace levels of the normal transcript are found.

In the first report [62], through in vitro studies using mouse *otc* minigenes transiently expressed in mouse hepatoma cells, the authors identified an ExSpeU1, which led to an increase in the levels of the correct transcript. The same was observed in vivo upon delivery via an AAV8 of the same ExSpeU1 to a mouse model carrying the c.386G>A variant (*spf*^ash^ mouse). In a second study [63], the same group dissected the molecular mechanisms mediating the differences in the pathogenic splicing patterns between mice and humans in the presence of the c.386G>A variant and how it can influence susceptibility to RNA-based therapies. Actually, they tested in vitro several engineered U1 snRNAs for the human sequence and none of those U1 snRNAs improved the selection of the authentic mutated 5′ss, because while decreasing exon skipping, they further promoted the usage of the adjacent cryptic 5′ss. The authors elegantly demonstrated that it can be attributed to the key role of subtle intronic changes downstream of the authentic 5′ss, which lead to differential binding of splicing factors and dictated the different splicing patterns between the two species. Altogether, these two studies highlight the importance of carefully investigating species-specific molecular mechanisms for translational purposes.

Another important challenge is related to the ineffectiveness, as reported in several studies [47,54,72], of the ExSpeU1 approach for mutations affecting the conserved GT dinucleotide at positions +1 and +2 of the 5′ss region, where the correction attempts also resulted in the activation of cryptic donor sites. Nevertheless, a pivotal study by Scalet and colleagues [17] targeting several splicing mutations in the *F9* gene causing Hemophilia B (Factor IX deficiency), proved that an ExSpeU1 can indeed rescue aberrant splicing caused by a variant located at the position +2 of the 5′ss. Initially, through minigene expression in mammalian cells, it was demonstrated that five naturally occurring variants in the 5′ss region of exon 3 (c.277+1G>A, c.277+1G>T, c.277+2T>C, c.277+2T>G and c.277+4A>G) induce complete exon 3 skipping. So, in an attempt to restore exon 3 definition a modified U1 snRNA totally complementary to the exon 3 5′ss and an ExSpeU1 targeting the downstream intron between positions +6 and +14 were designed and tested. As expected, for variant c.277+4A>G located in a less conserved position of the 5′ss, both modified U1 snRNA and ExSpeU1 promoted the inclusion of exon 3 (~60% of correct transcripts). Regarding the variants in the conserved +1 and +2 positions, the ExSpeU1 reduced the level of transcripts with exon 3 skipping but also led to the use of an exonic cryptic 5′ss located eight nucleotides upstream of the mutant 5′ss, which origins a frameshift transcript. However, surprisingly, the engineered ExSpeU1 also triggered the usage of the 5′ss affected by the c.277+2T>C mutation, allowing a significant recovery of correct transcripts (~20%). For both variants, the splicing rescue also produced a considerable increase in secreted FIX protein levels. Overall, this study broadens the spectrum of mutations, that are potentially amenable to ExSpeU1 treatment, thus adding a new perspective to the rescue of mutations that alter the conserved 5′ss GT dinucleotide.

### 3.3. When Modified U1 snRNAs Meet Other Therapeutic Molecules: Combined Therapeutic Strategies

While modified U1 snRNAs hold a therapeutic potential on their own, there are also approaches that combine modified U1 snRNAs with other therapeutic molecules. This is the case of a combined treatment for Bardet–Biedl syndrome recently developed by Breuel and colleagues [64] in which a modified U1 snRNA (to correct the exon skipping) and ASOs (to block intron retention) were used in an attempt to correct the effect of a mutation in the last base of exon 5 of the *BBS1* gene (c.479G>A), which leads to both exon 5 skipping and intron 5 retention. In fact, the team was able to prove that the combined use of these two splicing modulation molecules improved the efficacy of the splice correction and increased the amount of correctly spliced *BBS1* transcripts compared to single treatments.

However, this was not the only combined therapy including modified U1 snRNAs to be attempted. In fact, some years before, Schmid and co-workers [65] also used a combined treatment for the same disorder in which modified U1 snRNAs and U6 snRNAs with different compensatory mutations were tested for the correction of a mutation located at position +5 of *BBS1* gene intron 5 (see Table 1). Unlike the single treatment with a modified U1 snRNA, this combined treatment led to an almost complete correction of the splicing defect caused by the target mutation, showing that the sequence complementarity between U6 and the three splice donor site positions (+4, +5, and +6) was indeed relevant for the succeeded outcome. Nevertheless, the authors point out that the single use of modified U6 snRNA isoforms did not result in significant improvements.

A similar approach combining the modified U1 snRNAs and U6 snRNAs was also very recently developed by the same group [66] for the correction of the previously referred 5′ss *OPA1* mutation, c.1065+5G>A, which underlies a severe ocular disorder. These authors tested this combined therapy in a mouse model carrying the *Opa1* c.1065+5G>A 5′ss mutation (Opa1^enu/+^), which causes the same splicing defect observed in the patient’s fibroblasts. Briefly, three AAV2/8 vectors expressing different combinations of totally complementary (tc) or wild-type (wt) U1 and U6 sequences were produced: U1.wt/U6.wt, U1.tc/U6.wt and U1.tc/U6.tc. Importantly, the subretinal delivery of either the U1.tc/U6.wt or the U1.tc/U6.tc via AAV2/8 showed therapeutic efficacy and increased the *Opa1* wild-type transcripts expression in the eye. As no significant differences between the two treatments were observed on the transcript level, the effect on the protein was only tested using the U1.tc/U6.wt variant. The results showed a significant increase in Opa1 protein expression after treatment. Of notice, the expression of the engineered U1.tc/U6.wt did not induce obvious short-term side effects on the splicing of visual-associated genes as well on retinal morphology and functionality.

## 4. Factors to Consider in the Effectiveness and Use of Engineered U1 snRNA Vectors

Similar to any other therapeutic approach, the use of engineered U1 snRNAs also has some limitations. The fundamental limitation of both modified U1 snRNAs and ExSpeU1s is the possibility of unforeseen off-target effects in addition to a relatively low efficiency [35]. Theoretically, this seemed especially dangerous for the classical single base modified U1 snRNAs, which are designed to act over highly conserved regions. This meant that, ultimately, a modified U1 snRNA could be so similar to an endogenous U1 snRNA that it would bind to virtually any 5′ss. Plus, it would be more likely to activate cryptic splice-sites, thus affecting mRNA length and stability [18,70]. Intriguingly, however, recent in vivo assessments on the specificity of both modified U1 snRNAs [13] and ExSpeU1s [52,59] have provided similar results regarding their specificity. While somehow unexpected, these are encouraging results regarding the therapeutic potential of these molecules.

Overall, off-target effects are difficult to predict. That is why screening procedures must be undertaken as early as possible in the drug development pipeline in order to reduce the occurrence of non-specific bonds. In fact, to be able to predict the effect of any specific engineered U1 snRNA vector, experimental studies are required. Some relevant insights on the general effects of the classical modified U1 snRNAs and the novel ExSpeU1s have further highlighted their potential and specificity. Regarding the classical modified U1 snRNAs, when Balestra and co-workers [13] evaluated changes in the alternative splicing profile and in the global expression levels in liver specimens from mutation-specific FAH deficient mice treated with an AAV8-delivered modified U1 snRNA, negligible effects were observed. In fact, only a small proportion of the 13,000 investigated genes displayed an altered expression profile, and that percentage was even lower when only protein-coding genes were considered (one over- and three under-expressed genes). Similarly, recent transcriptome studies by Donadon et al. [52] and Romano and co-workers [59] have demonstrated that ExSpeU1s have high specificity for their target pre-mRNA, leading to minimal global changes in gene expression and splicing. Overall, the studies performed so far support the idea that the use of both modified U1 snRNAs and ExSpeU1s may be justified if the therapeutic benefits outweigh the drawbacks already indicated.

Last but not least, one of the most significant challenges in the therapeutic use of engineered U1 snRNA vectors, regardless of their generation, is their delivery to the target tissues/organs. This is actually a challenge faced by many other RNA-based therapeutics, such as small interfering RNAs (siRNAs) or even ASOs. In fact, even though successful delivery schemes have already been designed and implemented for some of these therapeutic molecules to reach a few specific organs (e.g.,: the eye, liver and even the central nervous system), systemic delivery is still an issue for many of them. Modified U1 snRNAs and ExSpeU1s in particular, are among those in need of additional delivery studies. Taking into account most of the data published so far, viral vectors will certainly be the first to reach clinical trials, as AAVs have been used as the principal delivery method in most of the in vivo studies developed. However, despite the changes made to these viral vectors, the potential for immune reactions to be triggered remains a significant limitation to their use and does raise a number of concerns. Still, it is worth mentioning that different AAV serotypes with specific tissue tropisms have been described, including serotypes that are able to transduce the blood-brain barrier (BBB) [73] a feature that makes them particularly suitable to deliver therapeutic agents for diseases that present neurological symptoms. Furthermore, AAV vectors have been shown to mediate long-term in vivo expression with little or no acute toxicity. These two observations are probably among the major reasons why AAV vectors have been extensively used not only in pre-clinical studies for different rare genetic diseases such as Lysosomal Storage Disorders, but also in numerous clinical trials for this group of diseases [74]. It should also be noted that the AAVs are nonpathogenic as they generally do not integrate into the host genome. Thus, in this respect, AAVs are safer than other types of viral vectors [75]. Overall, it is reasonable to assume that AAVs may as well be used for modified U1 snRNAs and the ExSpeU1s targeted delivery with equally promising results. Nevertheless, viral vector delivery is not the only possibility to take into account when designing a U1 snRNA-based therapeutic approach. Alternatives include delivering these molecules using nanoparticles, which will necessitate new research and development before this therapeutic strategy can be used in humans [18]. However, to the best of our knowledge, this type of approach has never been attempted for engineered U1 snRNAs.

While falling slightly out of the scope of this review, it should also be noticed that there is yet another U1 snRNA-related therapeutic approach, which can be attempted. It relies on the use of chimeric snRNA molecules carrying antisense sequences against the 3′ss of target exons for exon skipping purposes. This sort of approach was designed to counteract the fact that ASOs alone require periodic administration. To circumvent this problem, some teams made efforts to produce constructs, which would be able to express in vivo, in a stable fashion, large amounts of chimeric RNAs containing the therapeutic antisense sequences [76]. Those constructs are suitable for administration via traditional gene delivery vehicles, thus holding the potential to reduce the frequency of dosing [77].

To the best of our knowledge, this sort of approach was mainly tested as a possible therapeutic for Duchenne muscular dystrophy (DMD), which is probably the most well-known and in-depth studied muscular dystrophy of them all, in the RNA therapeutics field.

The rationale behind many of the studies that tested novel therapeutic approaches for DMD over the last decades was to convert severe DMD forms into milder ones through targeted exon skipping using antisense sequences against splice junctions and/or exonic splicing enhancers (ESEs) to induce the exclusion of certain exon(s) from the mature mRNA, while preserving the reading frame. Most teams are working with chemically modified ASOs, whose safety and efficacy are now largely documented, with several approved drugs reaching the market [78]. Others, however, have attempted to administer the therapeutic antisense sequences as part of a snRNA namely U1 [76,79,80,81,82] but also U7 [83,84,85,86,87]. Ultimately, one such approach may provide persistent expression, in vivo stability, and correct compartmentalization.

Indeed, chimeric U1 snRNA antisense molecules, i.e., ASOs raised in the U1 snRNA backbone, were shown to effectively induce the desired exon skipping not only in human DMD myoblasts [76,81,82] but also in DMD mouse models, namely the mdx mouse [79,80,88]. In both cases, the chimeric molecules were delivered as part of lentiviral or AAV vectors and provided high skipping activity and efficient rescue of dystrophin synthesis. Altogether, these results further highlight the therapeutic potential of U1 snRNAs, even though from a completely different perspective from that focused on this revision. This is particularly relevant since it has already been reported that the single-stranded 5′ terminus of U1 can be replaced by unrelated sequences as long as 50 nucleotides without noticeable effects not only over their stability but also on their capacity to assemble into snRNP particles. That makes it an effective vector for the stable expression of ASOs and for the inhibition of the splicing reaction [89]. Moreover, this approach can be appropriately designed to mediate either a single or multi-exon skipping [78,85]. To the best of our knowledge, however, none of the U1 snRNA antisense molecules has reached clinical trials. Still, there is an ongoing phase 1/2 clinical trial using a U7 snRNA-based approach for the correction of DMD exon 2 duplications (NCT04240314) [78].

## 5. Conclusions

Based on all the work here reviewed, there is a growing body of evidence on the potential of both modified U1 snRNAs and ExSpeU1s to mediate gene therapeutic correction of splicing defects underlying different genetic diseases. Remarkably, those positive results have been observed not only in vitro but also in vivo in different animal models. The first tests on animals started with modified U1 snRNAs and were evidenced by successful studies using ExSpeU1s.

It may be worth mentioning that a significant part of the studies relying on the use of both modified U1 snRNAs and ExSpeU1s was developed in the labs of Mirko Pinotti (University of Ferrara, Italy) and of Franco Pagani (Centre for Genetic Engineering and Biotechnology in Trieste, Italy) [12,13,38,44,48,51,58,59], where the whole pipeline to address their therapeutic potential in vitro and in vivo, seems to be well-implemented. However, it is important to stress that many other teams, in different labs, have been able to replicate the same sort of methods, achieving similar results for other disease-causing mutations [11,16,41,61,66]. This adds up to the overall therapeutic potential of engineered U1s, by demonstrating the replicability of the method and the robust correction levels it may promote. However, as with any other method, this approach also has some limitations. The most obvious and relevant one is the possibility of generating off-target effects. However, if this is a serious concern for modified U1 snRNAs, the emergence of the ExSpeU1s largely bypassed this issue by increasing its specificity and stability. Last but not least, the delivery of these molecules in vivo is a topic that also requires significant focus. In fact, targeted delivery of these molecules has been largely neglected as an object of study. However, this is a matter that requires further optimization and development to better test these molecules.

## Figures and Tables

**Table 1:** Summary table of engineered U1 small nuclear RNA (U1 snRNA) and combined therapeutic approaches developed for different human genetic diseases.

	Genetic Disease	Gene	Type of Study	Splicing Variants *	Reference
Modified U1 snRNAs	Neurofibromatosis type 1	*NF1*	in vitro	c.288+5G>C	[36]
	Coagulation factor VII deficiency	*F7*	in vitro	9726+5G>A (c.805+5G>A)	[37,38]
			in vivo	859+5G>A (c.805+5G>A)	[39]
	Retinitis pigmentosa	*RHO*	in vitro	c.936G>A	[40]
		*RPGR*	in vitro	c.1245+3A>T	[41]
	Bardet-Biedl syndrome	*BBS1*	in vitro	c.479G>A	[42]
	Propionyl-CoA carboxylase deficiency	*PCCA*	in vitro	c.1209+3A>G	[43]
	Tyrosinaemia type 1	*FAH*	in vitro	c.1062+5G>A	[44]
	Fanconi anemia	*FANCC*	in vitro	c.165+1G>T	[45]
	Mucopolysaccharidosis type IIIC	*HGSNAT*	in vitro	c.234+1G>A; c.633+1G>A; c.1542+4dupA	[11]
	Shwachman-Diamond syndrome	*SBDS*	in vitro	c.258+2T>C	[46]
	Aromatic L-Amino Acid Decarboxylase deficiency	*AADC*	in vitro	IVS6+4A>T	[16]
			in vivo	(c.714+4A>T)	
	ATP8B1 deficiency	*ATP8B1*	in vitro	c.2932-3C>A; c.2418+5G>A; c.279G>A; c.625_627+5delinsACAGTAAT	[10]
	CDKL5-deficiency	*CDKL5*	in vitro	c.99+1G>T	[47]
				c.99+5G>A	
				c.463+1G>A	
				c.744+1G>C	
				c.2376+5G>A	
	Tyrosinaemia type 1	*Fah*	in vivo	c.706G>A	[13]
Exon-Specific U1 snRNAs	Hemophilia B	*F9*	in vitro	c.392-9T>G; c.392-8T>G;	[48]
				c.519A>C; c.519A>G; c.519A>T	
				c.520G>T; c.520+1G>A; c.520+2T>C	
				p.V107V (c.459G>A);	[12]
				p.R116R (c.484C>A);	
				p.A118V (c.491C>T);	
				p. Q121H (c.501G>T)	
				c.277+1G>A; c.277+1G>T,	[17]
				c.277+2T>C; c.277+2T>G;	
				c.277+4A>G	
			in vitro	c.519A>C	[49]
			in vivo		
	Cystic fibrosis	*CFTR*	in vitro	c.1766+3A>G; c.1766+3A>C; c.1766+5G>A; c.1766G>T; c.1766G>A; p.A566T (c.1696G>A); p.Y577Y (c.1731C>T)	[48]
				711+3A>C (c.579+3A>C);	[50]
				711+3A>G (c.579+3A>G);	
				711+5G>A (c.579+5G>A);	
				1863C>T (c.1731C>T);	
				1898+3A>G (c.1766+3A>G);	
				2789+5G>A (c.2657+5G>A);	
				3120G>A (c.2988G>A);	
				TG13T3, TG13T5; TG12T5	
	Spinal muscular atrophy	*SMN2*	in vitro	c.840C>T	[48]
			in vitro	c.840C>T	[51,52,53]
			in vivo		
	ATP8B1 deficiency	*ATP8B1*	in vitro	c.625_627+5delinsACAGTAAT	[10]
	CDKL5-deficiency	*CDKL5*	in vitro	c.99+1G>T	[47]
				c.99+5G>A	
				c.463+1G>A	
				c.744+1G>C	
				c.2376+5G>A	
	Hemophilia A	*F8*	in vitro	c.602-32A>G; c.602-10T>G; c.669A>G; c.669A>T; c.670G>T; c.670+1G>T; c.670+1G>A; 670+2T>G; c.670+5G>A; c.670+6T>C	[54]
			in vitro	p.G2000A (c.5999G>C);	[55]
				p.Y2036Y (c.6108C>T);	
				p.N2038S (c.6113A>G);	
				c.6115+1G>A; c.6115+2T>C;	
				c.6115+3G>T; c.6115+4A>G;	
				c.6115+5G>A; c.6115+6T>A	
	Netherton syndrome	*SPINK5*	in vitro	c.891C>T	[56]
	Fanconi anemia	*FANCA*	in vitro	c.790C>T	[57]
	Familial dysautonomia	*ELP1*	in vitro	c.2204+6T>C	[58,59]
			in vivo		
	Autosomal dominant optic atrophy	*OPA1*	in vitro	c.1065+5G>A	[60]
	Phenylketonuria	*PAH*	in vitro	c.1199+17G>A; c. 1199+20G>C	[61]
	Shwachman-Diamond syndrome	*SBDS*	in vitro	c.258+2T>C	[46]
	Ornithine transcarbamylase deficiency	*Otc*	in vivo	c.386G>A	[62]
			in vitro		
		*OTC*	in vitro	c.386G>A	[63]
Combined therapies	Bardet-Biedl syndrome	*BBS1*	in vitro	c.479G>A	[64]
				Exon 5 5’ss (position +5) ^#^	[65]
	Autosomal dominant optic atrophy	*Opa1*	in vivo	c.1065+5G>A	[66]
	Hemophilia A	*F8*	in vitro	c.6115+5G>A	[55]

* All studied mutations were listed regardless of the outcome of the therapeutic approaches attempted. ^#^ Artificially generated variant that affects splicing but that has never been reported in a human patient so far.

## Data Availability

Not applicable.
